# miRNA Expression Associated with HbF in Saudi Sickle Cell Anemia

**DOI:** 10.3390/medicina58101470

**Published:** 2022-10-17

**Authors:** Cyril Cyrus, Chittibabu Vatte, Awatif Al-Nafie, Shahanas Chathoth, Mohammed S. Akhtar, Mohammed Darwish, Dana Almohazey, Saud H. AlDubayan, Martin H. Steinberg, Amein Al-Ali

**Affiliations:** 1 Department of Biochemistry, College of Medicine, Imam Abdulrahman Bin Faisal University, P.O. Box 1982, Dammam 31441, Saudi Arabia; 2 Department of Pathology, King Fahd Hospital of the University, College of Medicine, Imam Abdulrahman Bin Faisal University, Dammam 34445, Saudi Arabia; 3 Ministry of Health, Qatif Central Hospital, Qatif 32654, Saudi Arabia; 4 Department of Stem Cell Research, Institute for Research and Medical Consultations, Imam Abdulrahman Bin Faisal University, Dammam 31441, Saudi Arabia; 5 Department of Medical Oncology, Dana-Farber Cancer Institute, Boston, MA 02215, USA; 6 Department of Medicine, Division of Hematology and Medical Oncology, Boston University School of Medicine, Boston, MA 02118, USA

**Keywords:** miRNA, SCA, hemoglobinopathies, HbF, globin expression

## Abstract

*Background and Objectives*: Sickle cell anemia (SCA) is a hereditary monogenic disease due to a single β-globin gene mutation that codes for the production of sickle hemoglobin. Its phenotype is modulated by fetal hemoglobin (HbF), a product of γ-globin genes. Exploring the molecules that regulate γ-globin genes at both transcriptional and translational levels, including microRNA (miRNA), might help identify alternative therapeutic targets. *Materials and Methods*: Using next-generation sequencing we identified pre-miRNAs and mature miRNA expression signatures associated with different HbF levels in patients homozygous for the sickle hemoglobin gene. The involvement of identified miRNAs in potential SCD-related pathways was investigated with the DIANA TOOL and miRWalk 2.0 database. *Results*: miR-184 were most highly upregulated in reticulocytes. miR-3609 and miR-483-5p were most highly downregulated in sickle cell anemia with high HbF. miR-370-3p that regulates LIN28A, and miR-451a which is effective in modulating α- and β- globin levels were also significantly upregulated. miRNA targeted gene pathway interaction identified BCL7A, BCL2L1, LIN28A, KLF6, GATA6, solute carrier family genes and ZNF genes associated with erythropoiesis, cell cycle regulation, glycosphingolipid biosynthesis, cAMP, cGMP-PKG, mTOR, MAPK and PI3K-AKT signaling pathways and cancer pathways. *Conclusions*: miRNA signatures and their target genes identified novel miRNAs that could regulate fetal hemoglobin production and might be exploited therapeutically.

## 1. Introduction

Sickle cell anemia (SCA) is a result of homozygosity for a mutation in codon 6 of the β-globin gene (HBB; GAG→GTG) that leads to the production of sickle hemoglobin (HbS; glu7val; α2βS2). Fetal hemoglobin (HbF; α2γ2) encoded by HBG2 and HBG1 (HBG1/2) prevents the polymerization of deoxyHbS, the proximate cause of the phenotype of SCA, and therefore the major modulator of this disease [1,2].

Circulating hairpin and mature microRNAs (miRNAs) are small noncoding RNAs that modulate gene expression by inhibition of translation or stimulating degradation of gene transcripts. miRNAs are derived from distinct hairpin precursors (pre-miRNAs) and in contrast to mature miRNAs, profiling of pre-miRNAs is limited. These miRNA hairpin precursors, which fold into stem loop structures are ideal candidates for small molecule binding. A recent study reported knockdown of BCL11A using a miRNA-adapted short hairpin RNA for post-transcriptional silencing of BCL11A to treat SCD [3]. More than 1000 miRNAs are encoded within the human genome, and some were associated with HbF induction [4,5,6]. For example, BCL11A, a major suppressor HBG2/1 expression, is targeted by miR-210, Lin28B, miR-486-3p, miR-30a and Let-7 family [7,8,9,10]. Another HBG2/1 repressor, MYB, was targeted by miR-126 and miR-15a/-16-1 which increased HBG2/1 expression [11,12].

HbF levels vary 2 to 4-fold among the five common HBB haplotypes associated with the HbS gene. The Arab-Indian (AI) haplotype is associated with HbF that averages ~17% in adults compared with 5 to 7% in the Bantu, Benin and Cameroon haplotypes and 10% in the Senegal haplotype [2,13,14,15]. Even within the AI haplotype there is variation of HbF levels. The causes of high HbF in AI haplotype SCA are not fully explained [16]. To determine whether miRNAs have an association with HbF levels in AI haplotype SCA we explored the complete hairpin and mature miRNA expression profile of HbS homozygotes with “high” and “low” HbF levels.

## 2. Materials and Methods

### 2.1. Study Population and Samples

Participants aged from 3 to 42 years, 29 males (49%) and 30 females (51%), were clinically diagnosed by high performance liquid chromatography (HPLC) of hemoglobin. HbS homozygotes who were not having an acute vaso-occlusive episode or who were not currently on hydroxyurea, and normal hemoglobin controls were recruited from Qatif Central Hospital, Al-Qatif, Saudi Arabia. Blood samples (8–10 mL) were collected into EDTA tubes from 59 individuals that included 31 SCA patients and 28 healthy individuals without SCA. Blood counts were carried out by an automated clinical hematology analyzer (Sysmex, K-1000, Sysmex Corporation, Kobe, Japan), the percentage of HbS, HbA2 and HbF were measured by HPLC on the Bio-Rad Variant II Hb Testing System (Bio- Rad Laboratories, Hercules, CA, USA).

Ethical approval for the study was obtained from the Institutional Review Board Committee (IRB-2019-01-107) at Imam Abdulrahman bin Faisal University. The study was conducted according to the ethical principles of the Declaration of Helsinki and Good Clinical Practice Guidelines.

### 2.2. Magnetic Separation of CD71+ and CD235+ Cells

Peripheral blood mononuclear cells (PBMC) were isolated using density gradient Ficoll- Histopaque (Merck, St. Louis, MO, USA). PBMC were labeled with CD71 MicroBeads (Miltenyi Biotec, Bergisch Gladbach, Germany), which were used for the positive selection of human reticulocytes. The cell suspension was loaded onto a MACS^®^ Column which was placed in the magnetic field of a superMACS separator. The magnetically labeled CD71+ cells were retained on the column. After removal of the column from the magnetic field, the magnetically retained CD71+ cells were eluted as the positively selected cell fraction. Similarly, Glycophorin A+ (CD235+) cells were magnetically separated after labeling with Glycophorin-A MicroBeads (Miltenyi Biotec, Bergisch Gladbach, Germany). CD235a (Glycophorin A) MicroBeads were used for the positive selection of human erythroid cells as single-pass transmembrane glycoprotein is expressed on mature erythrocytes. These isolated cells were fluorescently stained with CD71-FITC and CD235-APC antibodies and confirmed by flow cytometry (Guava, Millipore, Burlington, MA, USA). Both these cell types were further processed for miRNA isolation.

### 2.3. RNA Extraction and Quantification

Total RNA was isolated using miRNeasy Mini kit (Qiagen, Hilden, Germany) in accordance with the manufacturer’s instructions to obtain 50 µL of extracted sample. The RNA isolation protocol utilizes QIAzol Lysis reagent, chloroform and ethanol. The final elution was with 50 µL of RNase-free water and was immediately quantified using the Qubit^®^ microRNA Assay Kits and the Qubit^®^ 4.0 Fluorometer (Thermo Fisher Scientific, Waltham, MA, USA). The quantified RNA was stored at −80 °C until library preparation.

### 2.4. miRNA Library Construction and Sequencing

Aliquots of 100 ng of miRNAs, derived from the above-mentioned procedure, were used for the preparation of miRNA libraries. Sequencing-ready cDNA libraries using the Small RNASeq Library Prep kit for Illumina (Lexogen, Austria) were prepared according to the manufacturer’s instructions. Multiplexing indices were introduced during the PCR amplification step to distinguish each sample after the pooling phase, allowing multiplexing of up to 96 libraries [17]. The library product was cleaned and concentrated with a magnetic bead-based purification protocol [18]. Lastly, the sequencing step was performed with NGS technologies using Illumina platform NextSeq 500 and the NextSeq 500/550 High Output v2.5 kit (150 cycles).

### 2.5. Bioinformatic Analysis of the Raw Sequencing Data

FASTQ files were generated via Illumina bcl2fastq2 (Version 2.17.1.14-http://support.illumina.Com/downloads/bcl-2fastq-conversion-software-v217.html accessed on 16 June 2021) starting from the raw sequencing reads produced by Illumina NextSeq sequencer. MiRNAs were mapped on miRbase hairpins using SHRiMP software. Differential expression analysis for miRNAs was performed with the R package DESeq2 (Ver 1.18.1) (R software, Vienna, Austria). MiRNAs with more than 5 counts were retained for further analysis. The miRNA expression is considered differentially significant if the log2(disease sample/healthy control) ≥ 1 and a False Discovery Rate (FDR) ≤ 0.1. Hence, a minimum |Log2FC| of 1 and an FDR lower than 0.1 as thresholds to differentially expressed genes to maximize the sensitivity of this analysis and to perform a massive screening and identification of candidate miRNAs. For comparative analysis, we dichotomized HbF levels into low (6.35 ± 3.57%) and high (21.31 ± 6.73%) groups [15,19].

### 2.6. Target Gene and Interaction Analysis

The involvement of identified miRNAs in potential SCD-related pathways (predicted target genes and predicted pathways) was investigated with the DIANA TOOL and miRWalk 2.0 database. Significantly differentially expressed miRNAs in SCA patients were considered for the analyses conducted on the mirWalk ver-2.0 to predict potential targets of these miRNAs [20]. Analysis was preferred for only validated results, using miRTarBase filter option and were compared using other popular algorithms, such as miRDB or Target Scan. DIANA-mirPath was used to hierarchical targeted genes clusters-based pathway identification utilizing the exact significance (*p*-value threshold set at 0.05) after FDR correction (Benjamini and Hochberg) and exclusion of pathways with few targeted genes nodes.

## 3. Results

Subject characteristics are shown in Table 1. Mean age in SCA was 22.45 ± 16.36 yrs. compared with 33.43 ± 13.2 yrs. in controls. Sixteen patients had low percentage of HbF (6.35 ± 3.57%) and 15 had high HbF% (21.31 ± 6.73%).

**Figure 1:**
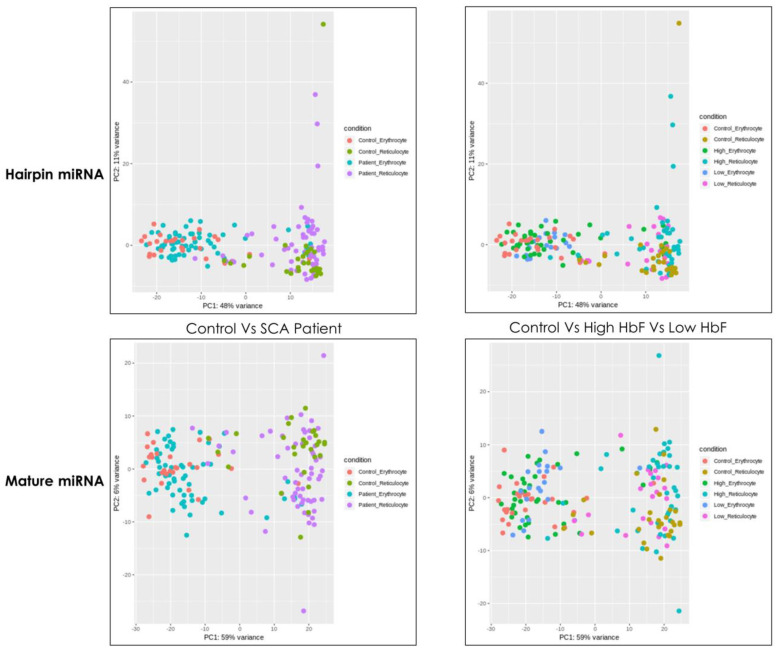
Principal Component Analysis (PCA) plot illustrating genetic relatedness among hairpin and mature miRNA in study cohort.

miR-184 was the most upregulated miRNA in the reticulocytes of the high HbF compared with the Low HbF cohort (fold = 2.83; *p* = 2.90 *×* 10^−03^; Supplementary Table S1). miR-4508 (fold = 4.36; *p* = 7 *×* 10^−08^), miR-451a (fold = 4.25; *p* = 3.6 *×* 10^−8^), miR-144 (fold = 2.78; *p* = 1.1 *×* 10^−4^) were upregulated in high HbF in reticulocytes compared with erythrocytes. In the high HbF cohort, the significantly downregulated miRNAs were mir-4655 (fold = −4.11; *p* = 1.70 *×* 10^−8^), mir-12118 (fold = −3.46; *p* = 4.40 *×* 10^−4^), mir-3125 (fold = −4.11; *p* = 5.90 *×* 10^−9^) and mir-7703 (fold = −3.49; *p* = 1.20 *×* 10^−3^) (Supplementary Table S2). miR-3609 (fold = −2.05; *p* = 2.50 *×* 10^−2^) in reticulocytes and miR-483-5p (fold = −3.6; *p* = 1.20 *×* 10^−2^) in erythrocytes were the most downregulated miRNA of high HbF compared with low HbF groups. The Principal Component Analysis (PCA) plot illustrating genetic relatedness among hairpin and mature miRNA is shown in Figure 1.

**Figure 2:**
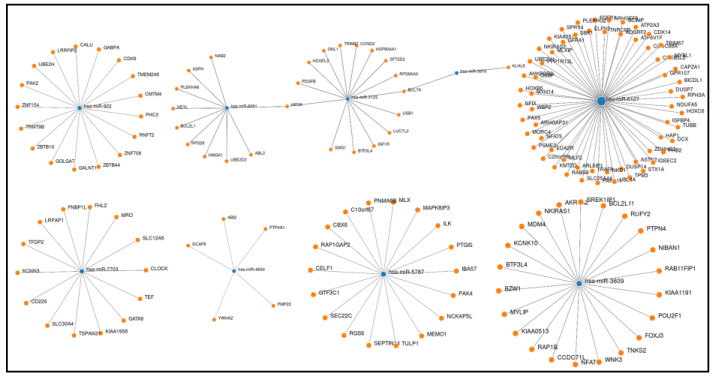
The node graphs of some dysregulated miRNA–target gene interaction associated with HbF level.

In gene pathway interaction assay, BCL7A, BCL2L1, LIN28A, KLF6, GATA6, SLC4A8, SLC12A5, SLC30A4, SLC30A6, ZNF154, ZNF706, ZNF618, ZNF655, ZNF789, ZNF394 and others were identified. The overall biological and functional role of these miRNAs–target genes in KEGG pathways were interpreted using DIANA tool (mirPath v.3). Genes targeted by the miRNAs were significantly enriched in erythropoiesis, cell cycle regulation, cAMP, cGMP-PKG signaling pathway, glycosphingolipid biosynthesis, mTOR, MAPK and PI3K-AKT signaling pathways. The dysregulated miRNAs and their target genes and associated pathways are listed in Table 2, while node graphs generated for these dysregulated miRNA–target gene interactions are shown in Figure 2.

## 4. Discussion

We studied miRNAs in Saudi HbS homozygotes with the AI haplotype from the South-Eastern Province, where α thalassemia is found in more than half of all people and where consanguinity is high, to see if additional insight might be gained into the mechanisms underlying HBG2/1 regulation in this unique haplotype. miRNAs previously associated with HbF expression in diverse SCA patients, including Lin28B, miR-486-3p, Let-7, miR-146a, miR-15a/-16-1, miR-126, miR-150, miR-199-5p, miR-221/-222, miR-23a, miR27a, miR-96, miR-26b, miR-144, miR-451 and miR-210. Let-7, mir-3609, miR-370-3p, miR-451a, mir-19b, mir-26a, and mir-15b were also dysregulated in AI haplotype SCA [21,22,23]. miRNA-370-3p (*p* = 7.90 × 10^−3^) was significantly upregulated in erythrocytes of the low HbF cohort. Gene target analysis showed that this miRNA regulates LIN28A. Lee et al. [7] assessed the capability of LIN28B to repress the expression of let-7 miRNA and determined that LIN28 overexpression or let-7 miRNA downregulation results in a 19–40% elevation in HbF levels in SCA. In addition, Lin28A obstructs the pre-let-7 processing by Dicer via recruiting terminal uridylyl transferase 4 [24]. Hence, miR-370-3p could be a novel target to regulate LIN28 for elevated HbF production.

Similarly, miR-451a was upregulated in reticulocytes (*p* = 7.10 × 10^−4^). Expression of miRNA-451 is elevated during erythroid cell differentiation. Fang and Bartel [25] reported that miR-451, which derives from a hairpin with a suboptimal terminal loop and a suboptimal stem length, accumulates 40-fold higher levels when clustered with a helper hairpin. miR-451 was upregulated in reticulocytes by approximately 2.5-fold in the high HbF cohort. miRNA-451 forms a cluster with miRNA-144 and regulates the expression of many genes necessary for erythropoiesis. In 6 SCA patients with HbF 23.5 ± 2.1% and 6 patients with HbF 3.41 ± 1.0%, all with undetermined haplotypes, miRNA-144 expression in reticulocytes was upregulated 8-fold in low vs. high HbF patient groups [26]. miR-144 and its mature forms were significantly dysregulated in the reticulocytes of high HbF cohort compared with erythrocytes. Functional analysis of normal and sickle erythroid progenitors showed that the miRNA-144 antagomir can facilitate HbF production while increasing NRF2 expression [26]. Among eight adult patients with the Bantu HbS haplotype, 22 of 798 miRNAs were differentially expressed with 13 associated with genes that regulate *HBG2/1* expression like *BCL11A* (miR-148b-3p, miR-32-5p, miR-340-5p, and miR-29c-3p), *MYB* (miR-105-5p), and *KLF3* (miR-106b-5), and SP1 (miR-29b-3p, miR-625-5p, miR-324-5p, miR-125a-5p, miR-99b-5p, miR-374b-5p, and miR-145-5p) [23].

The zinc finger proteins, *ZNF802* and *ZNF410* are HbF gene repressors. Induced deficiency of ZNF802 resulted in an increase in HbF to 35.0  ±  3.5% [27]. Knockout of *ZNF410* reduced Chromodomain Helicase DNA Binding Protein 4 (CHD4) levels by 60%, enough to substantially derepress HbF [28]. *ZNF154*, *ZNF706*, *ZNF655*, *ZNF789*, *ZNF394* and *ZNF618* were among the miRNA targets of the present study. Similarly, other than the well-studied *BCL11A, BCL7A* and *BCL2L1* were also among the target genes in the present study. *BCL2L1* has been associated with HbF gene expression in patients with SCA, where overexpression of *BCL2L1* resulted in increased *HBG2*/1 expression fourfold and F-cells by 13% [29]. Likewise, other than the known *KLF1*, an interaction between *KLF6*, a new potential regulator of erythropoiesis and miR-2355-5p was identified, and its downregulation significantly raised γ-globin mRNA [30]. miR-181c-5p targeted *KLF6*, and the significantly downregulated miR-3609 targeted the binding site of *KLF6* [31].

Hematopoietic stem cell differentiation is associated with activation of multiple signaling pathways including MAPK and PI3K [32,33,34]. We found novel miRNAs, miR-5787, miR-3609 and miR-483-5p associated with these signaling pathways might be potentially exploited as therapeutic targets. Histone deacetylase inhibitors like Apicidin and Tricostatin, increase H3 and H4 acetylation in the region adjacent to the HBG2/1 promoters [35] and induce γ-globin gene expression [36] by activating MAPK signaling pathway.

## 5. Conclusions

miRNA signatures and their target gene-pathway analysis identified novel miRNAs that could regulate fetal hemoglobin production and might be exploited therapeutically. Additional studies in erythroid cell lines and primary erythroid progenitor cells are needed to define how any of the identified miRNAs might modulate *HBG2/1* expression and the accumulation of HbF and if some of the HbF-associated miRNAs can account for the high levels of HbF in AI haplotype SCA.

## Figures and Tables

**Table 1:** Baseline characteristics and hematological parameters.

	Control	HbS Homozygote	Low HbF	High HbF	Control vs. SCA	Low HbF vs. High HbF
	(N = 28)	(N = 31)				
	Mean ± SD	Mean ± SD	Mean ± SD	Mean ± SD	*p* Value	*p* Value
**Age (Y)**	33.43 ± 13.2	22.45 ± 16.36	17.34 ± 16.05	27.25 ± 15.64	0.01	0.092
**HbF (%)**	0.83 ± 0.32	14.98 ± 9.34	6.35 ± 3.57	21.31 ± 6.73	<0.0001	<0.0001
**HbA_2_ (%)**	3.54 ± 1.03	3.03 ± 0.5	2.91 ± 0.6	3.2 ± 0.29	0.12	0.193
**RBC (10^3^/mm^3^)**	5.79 ± 0.92	4.13 ± 0.74	3.9 ± 0.61	4.44 ± 0.83	<0.0001	0.097
**Hb (g/dL)**	12.04 ± 1.58	10.65 ± 1.6	10.92 ± 1.42	10.3 ± 1.82	0.09	0.366
**Hematocrit (%)**	40.18 ± 5.98	31.49 ± 5.24	31.97 ± 4.08	30.91 ± 6.56	<0.0001	0.648
**MCV (fL)**	69.92 ± 8.72	78.17 ± 10.67	82.86 ± 10.14	72.55 ± 8.7	0.12	0.019
**MCH (pg)**	21.06 ± 2.97	26.42 ± 3.95	28.01 ± 3.43	24.51 ± 3.83	0.01	0.035
**MCHC (g/dL)**	30.04 ± 1.34	33.68 ± 1.56	33.9 ± 1.22	33.42 ± 1.93	<0.0001	0.485
**Platelets (10^3^/mm^3^)**	393.2 ± 72.9	271.7 ± 112.5	229.33 ± 75.03	322.6 ± 131.82	0.03	0.05

**Table 2:** Dysregulated miRNA, target gene interaction and associated pathways.

Cohort	Dysregulated miRNA	Target Genes	Targeted Pathways
Low HbF vs. High HbF upregulated	miR-184	BIN3, LRRC8A	Arrhythmogenic right ventricular cardiomyopathy (ARVC) Adherens junction Adrenergic signaling in cardiomyocytes Oocyte meiosis Signaling pathways regulating pluripotency of stem cells Thyroid hormone synthesis Glioma Choline metabolism in cancer Pancreatic cancer Hippo signaling pathway GnRH signaling pathway Oxytocin signaling pathway cGMP-PKG signaling pathway Mucin type O-Glycan biosynthesis Glycosphingolipid biosynthesis-ganglio series Acute myeloid leukemia Axon guidance Inflammatory mediator regulation of TRP channels Insulin secretion Salivary secretion Fatty acid elongation
	miR-3125	BCL7A, BTF3L4, CCND2, GNL1, HDGFL3, HIF3A, HSP90AA1, IGF1R, LUC7L2, PDGFB, RPS6KA3, RPS6KA3, SFT2D2, SMG1, TRIM67, USB1	
	miR-3169	TMCC1, TTL	
	miR-3668	BTBD3	
	miR-3976	BCL7A, KLHL8	
	miR-4261	NFAT5	
	miR-4327	FNDC3B, TMEM41B, TP53INP1	
	miR-4654	ABI2, DCAF8, PMP22, PTP4A1, YWHAZ	
	miR-4754	ARMCX6	
	miR-5787	C10orf67, CBX6, CELF1, GTF3C1, IBA57, ILK, MAPK8IP3, MEMO1, MLX, NCKAP5L, PAK4, PNMA8B, PTGIS, RAP1GAP2, RGS6, SEC22C, SEPTIN14, TULP1	
	miR-6081	ABL2, ASPH, BCL2L1, HEYL, HIF3A, HMGA1, NAB2, PLEKHA6, RPS28, UBE2D3	
	miR-662	MCFD2	
	miR-7703	CD226, CLOCK, FHL2, FNBP1L, GATA6, KCNN3, KIAA1958, LRPAP1, MRO, SLC12A5, SLC30A4, TEF, TFDP2, TSPAN31	
	miR-922	CALU, CDK6, CMTM4, GABPA, GALNT1, GOLGA7, LRRFIP2, PAK2, PHC3, RNFT2, TMEM248, TRMT9B, UBE2H, ZBTB10, ZBTB44, ZNF154, ZNF706	
Low HbF vs. High HbF downregulated	miR-3609	AKR7A2, BCL2L11, BTF3L4, BZW1, CCDC71L, FOXJ3, KCNK10, KIAA0513, KIAA1191, MDM4, MYLIP, NFAT5, NIBAN1, NKIRAS1, POU2F1, PTPN4, RAB11FIP1, RAP1B, RUFY2, SREK1IP1, TNKS2, WNK3, ZNF655, ZNF789, ZNF394	Drug metabolism-cytochrome P450 Transcriptional misregulation in cancer Ubiquitin mediated proteolysis Lysine degradation PI3K-Akt signaling pathway Mucin type O-Glycan biosynthesis Neurotrophin signaling pathway
	miR-483-5p	MAPK3	
Control vs. High HbF upregulated	miR-382-3p	ATP13A3, CCNT1M, KLHL29, METAP1, SLC4A8	Glycosaminoglycan biosynthesis - heparan sulfate/heparin Ubiquitin mediated proteolysis Gap junction Colorectal cancer Basal cell carcinoma Valine, leucine and isoleucine biosynthesis mTOR signaling pathway Tyrosine metabolism Long-term potentiation Endocytosis TGF-beta signaling pathway RNA degradation Osteoclast differentiation
	miR-451a	VAPA	
Control vs. High HbF downregulated	miR-184	CRISPLD2, EPB41L5, FBXO28, SF1	Lysine degradation Pentose phosphate pathway Caffeine metabolism Biosynthesis of unsaturated fatty acids Taurine and hypotaurine metabolism
	miR-542-3p	ASRGL1, EDEM3, GPATCH2L, GUCY1A2, HIPK3, MAP7, PLEKHM3, SAMD12, SLC30A6, VAPB, ZNF618	
Control vs. Low HbF upregulated	miR-134-5p	ANGPTL4	Estrogen signaling pathway Purine metabolism Rap1 signaling pathway Long-term potentiation Proteoglycans in cancer Glioma Thyroid cancer Acute myeloid leukemia Prolactin signaling pathway Non-small cell lung cancer
	miR-181c-5p	CPEB4, DDX3X, ETS1, KLF6, MECP2, MTX3, NAA50, OSBPL3, RCOR1, RPS6KA3, TRIM2	
	miR-370-3p	CYB561D1, LIN28A, NF1, NSUN4, PARVB, TGFBR2	

## Data Availability

Not applicable.

## Supplementary Materials

The following supporting information can be downloaded at: https://www.mdpi.com/article/10.3390/medicina58101470/s1, Supplementary Table S1: List of upregulated miRNAs with more than 1.5-fold change with HbF level in SCA cohort; Supplementary Table S2: List of downregulated miRNAs with more than 1.5-fold change with HbF level in SCA cohort.
