# Supplementary material for: miRNA Expression Associated with HbF in Saudi Sickle Cell Anemia
Source: Medicina (Kaunas). 2022 Oct 17;58(10):1470. doi: 10.3390/medicina58101470 (PMC9611475; doi:10.3390/medicina58101470)
Supplement: Supplementary file 1 [file medicina-58-01470-s001.zip › medicina-1937354-supplementary.pdf]

Supplementary Table S1. List of upregulated miRNAs with more than 1.5-fold change with HbF level in SCA cohort

Cell Type	miRNA/pre-miRNA	Gene ID	Fold Change (log_2)	P value
**Control vs. SCA-High HbF**				
Reticulocyte	Hairpin miRNA	hsa-mir-1296	1.52	4.40E-02
Reticulocyte	Mature miRNA	hsa-miR-382-3p	1.83	9.70E-02
Reticulocyte	Mature miRNA	hsa-miR-451a	2.13	1.40E-02
Reticulocyte	Hairpin miRNA	hsa-mir-548al	1.54	4.00E-02
Erythrocyte	Mature miRNA	hsa-mir-6127	1.59	1.30E-03
Reticulocyte	Mature miRNA	hsa-miR-641	1.6	1.30E-02
Reticulocyte	Hairpin miRNA	hsa-mir-758	1.64	9.60E-02
**Control vs. SCA-Low HbF**				
Reticulocyte	Hairpin miRNA	hsa-mir-1296	1.74	5.80E-02
Erythrocyte	Mature miRNA	hsa-miR-134-5p	1.73	5.70E-02
Erythrocyte	Mature miRNA	hsa-miR-181c-5p	2.72	1.40E-10
Erythrocyte	Hairpin miRNA	hsa-mir-3143	2.19	2.20E-03
Erythrocyte	Hairpin miRNA	hsa-mir-3146	2.32	2.50E-02
Erythrocyte	Mature miRNA	hsa-miR-3200-5p	1.62	4.20E-02
Erythrocyte	Mature miRNA	hsa-miR-370-3p	1.88	7.90E-03
Erythrocyte	Mature miRNA	hsa-miR-4441	1.93	4.80E-02
Erythrocyte	Mature miRNA	hsa-miR-451a	1.7	6.10E-02
Erythrocyte	Hairpin miRNA	hsa-mir-4646	1.84	8.50E-02
Erythrocyte	Mature miRNA	hsa-miR-4646-5p	2.09	4.80E-02
Erythrocyte	Hairpin miRNA	hsa-mir-5090	2.04	1.60E-02
Erythrocyte	Hairpin miRNA	hsa-mir-636	1.67	1.90E-02
Erythrocyte	Hairpin miRNA	hsa-mir-658	2.61	2.00E-03
Erythrocyte	Mature miRNA	hsa-miR-6734-5p	1.5	4.30E-02
Erythrocyte	Hairpin miRNA	hsa-mir-6747	1.61	7.00E-02
**SCA - Low HbF vs. High HbF**				
Reticulocyte	Hairpin miRNA	hsa-mir-12118	2.55	8.20E-02
Reticulocyte	Mature miRNA	hsa-miR-184	2.83	2.90E-03
Reticulocyte	Hairpin miRNA	hsa-mir-219a-1	2.47	8.30E-04
Reticulocyte	Hairpin miRNA	hsa-mir-3125	3.18	8.30E-04
Reticulocyte	Hairpin miRNA	hsa-mir-3168	2.79	8.50E-03
Reticulocyte	Hairpin miRNA	hsa-mir-3169	2.62	5.10E-03
Reticulocyte	Hairpin miRNA	hsa-mir-3180-5	2.58	9.70E-02
Reticulocyte	Hairpin miRNA	hsa-mir-3668	2.64	9.10E-02
Reticulocyte	Hairpin miRNA	hsa-mir-3976	2.51	5.90E-02
Reticulocyte	Hairpin miRNA	hsa-mir-4261	2.71	8.30E-04
Reticulocyte	Hairpin miRNA	hsa-mir-4327	2.79	7.10E-02
Reticulocyte	Hairpin miRNA	hsa-mir-4654	2.63	2.90E-02
Reticulocyte	Hairpin miRNA	hsa-mir-4655	2.72	8.70E-03
Reticulocyte	Hairpin miRNA	hsa-mir-4754	2.78	8.70E-03
Reticulocyte	Hairpin miRNA	hsa-mir-5787	2.69	9.70E-02
Reticulocyte	Hairpin miRNA	hsa-mir-6081	3.25	3.50E-02
Reticulocyte	Hairpin miRNA	hsa-mir-662	2.83	9.10E-02
Reticulocyte	Hairpin miRNA	hsa-mir-6813	3.49	7.00E-05
Reticulocyte	Hairpin miRNA	hsa-mir-6885	2.72	2.50E-02
Reticulocyte	Hairpin miRNA	hsa-mir-7703	2.65	9.10E-02
Reticulocyte	Hairpin miRNA	hsa-mir-922	2.66	9.70E-02

Supplementary Table S2. List of downregulated miRNAs with more than 1.5-fold change with HbF level in SCA cohort

Cell Type	miRNA/pre-miRNA	Gene ID	Fold Change (log_2)	P value
**Control vs. SCA High HbF**				
Reticulocyte	Mature miRNA	hsa-miR-184	-3.4	2.70E-08
Reticulocyte	Mature miRNA	hsa-miR-3138	-2.62	3.60E-02
Reticulocyte	Mature miRNA	hsa-miR-510-5p	-3.05	1.40E-02
Reticulocyte	Hairpin miRNA	hsa-mir-12118	-3.46	4.40E-04
Reticulocyte	Mature miRNA	hsa-miR-4725-3p	-2.57	7.40E-02
Reticulocyte	Hairpin miRNA	hsa-mir-33b	-3.28	7.80E-04
Reticulocyte	Mature miRNA	hsa-miR-1229-5p	-2.07	9.20E-02
Reticulocyte	Mature miRNA	hsa-miR-3192-5p	-1.95	3.80E-02
Reticulocyte	Hairpin miRNA	hsa-mir-6075	-3.66	1.90E-04
Reticulocyte	Hairpin miRNA	hsa-mir-7703	-3.49	1.20E-03
Reticulocyte	Hairpin miRNA	hsa-mir-3125	-4.11	5.90E-09
Reticulocyte	Hairpin miRNA	hsa-mir-5787	-3.39	4.10E-03
Reticulocyte	Mature miRNA	hsa-miR-4755-5p	-1.57	9.80E-02
Reticulocyte	Mature miRNA	hsa-miR-6880-5p	-1.85	1.70E-03
Reticulocyte	Hairpin miRNA	hsa-mir-4261	-3.96	1.90E-11
Reticulocyte	Mature miRNA	hsa-miR-5100	-1.61	5.80E-02
Reticulocyte	Mature miRNA	hsa-miR-542-3p	-1.84	7.60E-03
Reticulocyte	Mature miRNA	hsa-miR-1260a	-1.56	7.60E-03
Reticulocyte	Mature miRNA	hsa-miR-4454	-2.14	9.00E-03
Reticulocyte	Mature miRNA	hsa-miR-6747-3p	-1.97	5.80E-02
Reticulocyte	Mature miRNA	hsa-miR-4771	-2.41	1.40E-02
Reticulocyte	Mature miRNA	hsa-miR-4485-3p	-1.81	6.40E-02
Reticulocyte	Hairpin miRNA	hsa-mir-4655	-4.11	1.70E-08
Reticulocyte	Mature miRNA	hsa-miR-6820-3p	-1.55	1.60E-02
Reticulocyte	Hairpin miRNA	hsa-mir-5691	-2.86	3.40E-02
Erythrocyte	Mature miRNA	hsa-miR-4454	-1.89	7.40E-02
Reticulocyte	Hairpin miRNA	hsa-mir-8060	-3.19	3.70E-09
Reticulocyte	Hairpin miRNA	hsa-mir-1282	-3.19	4.70E-03
Reticulocyte	Hairpin miRNA	hsa-mir-6813	-3.25	1.40E-06
Reticulocyte	Hairpin miRNA	hsa-mir-5188	-3.16	1.90E-03
Reticulocyte	Hairpin miRNA	hsa-mir-650	-2.97	4.40E-04
Reticulocyte	Hairpin miRNA	hsa-mir-3137	-2.84	4.40E-02
Erythrocyte	Mature miRNA	hsa-miR-4497	-2.77	7.10E-02
Erythrocyte	Hairpin miRNA	hsa-mir-3609	-2.78	6.70E-04
Erythrocyte	Hairpin miRNA	hsa-mir-4485	-2.1	3.90E-03
Reticulocyte	Hairpin miRNA	hsa-mir-561	-3.2	4.40E-04
Reticulocyte	Hairpin miRNA	hsa-mir-1199	-2.97	3.40E-02
Reticulocyte	Hairpin miRNA	hsa-mir-6081	-2.97	1.50E-02
Erythrocyte	Mature miRNA	hsa-miR-4485-3p	-2.25	7.60E-02
Erythrocyte	Hairpin miRNA	hsa-mir-4497	-4.05	4.20E-08
Erythrocyte	Hairpin miRNA	hsa-mir-5588	-1.76	2.30E-02
Reticulocyte	Hairpin miRNA	hsa-mir-4784	-3.15	1.80E-03
Reticulocyte	Hairpin miRNA	hsa-mir-564	-3.01	1.50E-02
Erythrocyte	Hairpin miRNA	hsa-mir-12136	-1.95	6.70E-04
**Control vs. SCA-Low HbF**				
Erythrocyte	Hairpin miRNA	hsa-mir-7-1	-1.53	1.40E-02
Erythrocyte	Hairpin miRNA	hsa-mir-577	-1.62	9.70E-02
Erythrocyte	Hairpin miRNA	hsa-mir-1246	-1.98	1.90E-02

Erythrocyte	Hairpin miRNA	hsa-mir-3609	-1.98	4.00E-02
Erythrocyte	Hairpin miRNA	hsa-mir-574	-1.62	1.00E-05
Erythrocyte	Mature miRNA	hsa-miR-1249-3p	-1.62	4.20E-02
Erythrocyte	Mature miRNA	hsa-miR-12136	-1.78	4.90E-02
Erythrocyte	Mature miRNA	hsa-miR-6131	-1.98	1.40E-10
Erythrocyte	Mature miRNA	hsa-miR-1261	-1.63	1.90E-03
Erythrocyte	Mature miRNA	hsa-miR-1260a	-1.61	3.20E-03
Erythrocyte	Mature miRNA	hsa-miR-3175	-1.76	1.80E-02
Erythrocyte	Mature miRNA	hsa-miR-6880-5p	-1.75	1.40E-03
Reticulocyte	Mature miRNA	hsa-miR-1260a	-1.59	1.20E-02
Reticulocyte	Hairpin miRNA	hsa-mir-650	-2.26	4.30E-02
Reticulocyte	Hairpin miRNA	hsa-mir-1290	-2.98	8.40E-03
Reticulocyte	Hairpin miRNA	hsa-mir-4326	-1.67	5.80E-02
Reticulocyte	Hairpin miRNA	hsa-mir-182	-1.74	7.60E-04
Reticulocyte	Hairpin miRNA	hsa-mir-5588	-1.87	2.20E-02
Reticulocyte	Hairpin miRNA	hsa-mir-1246	-2.29	6.50E-03
Reticulocyte	Hairpin miRNA	hsa-mir-873	-2.52	5.80E-02
Reticulocyte	Hairpin miRNA	hsa-mir-4497	-2.25	2.20E-02
Reticulocyte	Hairpin miRNA	hsa-mir-4512	-1.53	8.40E-02
Reticulocyte	Hairpin miRNA	hsa-mir-3609	-2.66	2.00E-04
Reticulocyte	Hairpin miRNA	hsa-mir-1270	-1.55	2.20E-02
Erythrocyte	Mature miRNA	hsa-miR-483-5p	-3.66	2.60E-05
Reticulocyte	Hairpin miRNA	hsa-mir-9718	-1.79	5.80E-02
Reticulocyte	Hairpin miRNA	hsa-mir-4443	-1.8	6.40E-03
Erythrocyte	Mature miRNA	hsa-miR-4454	-2.61	3.70E-04
Erythrocyte	Hairpin miRNA	hsa-mir-4497	-3.48	1.00E-05
**SCA - Low HbF vs. High HbF**				
Reticulocyte	Hairpin miRNA	hsa-mir-3609	-2.05	2.50E-02
Erythrocyte	Mature miRNA	hsa-miR-483-5p	-3.6	1.20E-02
